# Associations between individual variations in visual attention at 9 months and behavioral competencies at 18 months in rural Malawi

**DOI:** 10.1371/journal.pone.0239613

**Published:** 2020-10-01

**Authors:** Juha Pyykkö, Ulla Ashorn, Eletina Chilora, Kenneth Maleta, Per Ashorn, Jukka M. Leppänen

**Affiliations:** 1 Center for Child Health Research, Faculty of Medicine and Health Technology, Tampere University, Tampere, Finland; 2 Department of Public Health, School of Public Health and Family Medicine, College of Medicine, University of Malawi, Blantyre, Malawi; 3 Department of Paediatrics, Tampere University Hospital, Tampere, Finland; 4 Infant Cognition Laboratory, Center for Child Health Research, Faculty of Medicine and Health Technology, Tampere University, Tampere, Finland; SWPS University of Social Sciences and Humanities, POLAND

## Abstract

Theoretical and empirical considerations suggest that individual differences in infant visual attention correlate with variations in cognitive skills later in childhood. Here we tested this hypothesis in infants from rural Malawi (*n* = 198–377, depending on analysis), who were assessed with eye tracking tests of visual orienting, anticipatory looks, and attention to faces at 9 months, and more conventional tests of cognitive control (A-not-B), motor, language, and socioemotional development at 18 months. The results showed no associations between measures of infant attention at 9 months and cognitive skills at 18 months, either in analyses linking infant visual orienting with broad cognitive outcomes or analyses linking specific constructs between the two time points (i.e., switching of anticipatory looks and manual reaching responses), as correlations varied between -0.08 and 0.14. Measures of physical growth, and family socioeconomic characteristics were also not correlated with cognitive outcomes at 18 months in the current sample (correlations between -0.10 and 0.19). The results do not support the use of the current tests of infant visual attention as a predictive tool for 18-month-old infants’ cognitive skills in the Malawian setting. The results are discussed in light of the potential limitations of the employed infant tests as well as potentially unique characteristics of early cognitive development in low-resource settings.

## Introduction

The ability to control visual attention emerges early in development, allowing infants to turn their gaze into salient objects in their visual environment (e.g., faces) and anticipate predictable visual events. The study of infants’ attentional abilities and looking times has a long history in developmental psychology [1], and has recently become more efficient, precise, and objective with the advent of remote eye tracking technologies [2]. Because looking behavior can be studied with eye tracking long before children reach the age at which they can be instructed to perform more complex cognitive tasks [2, 3], there have been hopes that the measurement of infant attentional skills may provide a way to investigate individual variations at an earlier age than is possible with traditional cognitive tests (e.g. [4]). This possibility is supported by theoretical considerations and empirical, longitudinal data.

*First*, the speed of simple sensory-motor responses, such as the orienting of attention to the abrupt onset of a stimulus, may provide a proxy for the overall “integrity” of the central nervous system and, by consequence, efficiency of information processing across multiple domains of cognitive function [5]. Consistent with this possibility, longitudinal data from studies conducted in the USA have shown that faster speed of oculomotor orienting responses to pictures on computer screen in infancy (e.g., 3.5–7 months) is correlated with higher IQ at 4 years [6] and executive functioning at 11 years [7]. Whilst the exact mechanisms of these associations are not known, one possibility is that infants’ capacities are predictive because individual differences in cognitive function tend to covary across domains and even across domains that become measurable at different ages (e.g., motor, cognitive, and language abilities). This line of reasoning is also supported by a recent longitudinal cohort study showing that developmental delays manifest in fine motor processes during the first year and across motor, visual, and language abilities during the second and third year of life [8].

*Second*, infants’ capacities may predict later cognitive outcomes given overlap in the neural mechanisms of cognitive processes. Frontoparietal networks may, for example, be important for attentional orienting, which develops during the first year of life, and executive functions, which develops more slowly throughout early childhood [9]. Overlap in neural bases may explain findings from the USA showing that attentional orienting at 5 months (i.e., median duration and number of looks at dynamic stimuli) predicts executive function at 10 to 36 months of age (e.g. [9–12]). It may even be possible that the same neurocognitive skill (e.g., cognitive control) first manifests in looking behavior (given early maturation of oculomotor control) and later in manual, reaching responses (after reaching matures during the second year of life [13]). For example, infants begin to anticipate visual targets during the first year and can “update” these responses after a change in target location [14] (although, see [15]). The cognitive processes underlying this capacity may be the same as those involved in more complex manifestation of cognitive control later in life (e.g., searching for the reward from a new location in the A-not-B task [16]).

Moving from purely cognitive processes to early markers of social cognitive function, past research has shown that increased attention to faces in infancy is associated with empathy-related and prosocial behaviors later in childhood [17, 18]. Attention to faces in infancy may be a prerequisite for learning about others and, ultimately, learning empathic abilities. Alternatively, there may be genetic or environmentally caused variations that affect the expression of social behaviors in children, including attentiveness to faces early in life and empathy-related traits, and explain the co-variations among measures of these constructs. In either case, attention to faces during the second half of the first year may provide a useful marker of infants’ early social development.

The existing data are, therefore, consistent with the possibility that infants’ attentional functions provide a potentially useful indicator of early cognitive and social development. As yet, the studies supporting this possibility are, however, limited to high-income settings in Europe and North America and there is a paucity of data on the predictive value of various attentional measures in low-resource settings where a significant proportion of the infants grow up [19]. Recent studies have shown that it is feasible to use eye tracking technologies to assess infants’ attentional abilities in heterogeneous environments, including low socioeconomic environments in London [20, 21], rural villages in Malawi [22], Gambia [23], and Vanuatu [24]. Results from these studies point to the existence of similar attentional capacities in infants across various rearing environments. What is not known, however, is whether infant attentional capacities are predictive of long-term cognitive development in low-resource settings. This question is of importance not only for expanding the research on early cognitive development to populations that have been under-represented in developmental psychology [19], but also for examining the possibility that eye tracking measures may provide much needed early outcomes of cognitive development for studies conducted in these environments.

In the current study with Malawian children, we examined associations between individual variations in tests of visual attention at 9 months of age and a range of development tests (vocabulary, socioemotional skills, motor development, executive function) at 18 months of age. We focused on three domains of early attention: visual search for a target [25, 26], anticipatory attention shifts [14], and attention to faces (e.g. [27–29]). Individual variations in these skills may start to emerge as early as 3–7 months of age [6, 7, 29, 30], and show low to moderate stability over a short test-retest interval between 5 and 7, 7 and 9, as well as 9 and 11.5 months [31, 32]. Accordingly, in the current study, we tested whether the speed of visual orienting at 9 months [31] correlates with cognitive outcomes across domains at 18 months, and whether there are links between specific cognitive processes in infancy (i.e., switching of anticipatory looks, attention to faces) and putatively overlapping cognitive or behavioral processes later in childhood (i.e., performance of A-not-B task, socioemotional behavior, respectively). As a secondary aim, we replicated previous analyses examining how growth and psychosocial risk factors relate to cognitive outcomes at 18 months (e.g. [33–42]). The cognitive outcomes were assessed at 18 months of age in the current study as this is the age when individual differences become visible across domains (i.e., motor, language, social-emotional, executive functions, see [8]; see also [9, 10]) and have been studied through observational and parent report methods, also in the target population in Malawi [43–45].

## Materials and methods

### Participants

444 infants without known congenital malformation, severe illness, or visual impairment were enrolled after birth into a prospective cohort study in Lungwena and Malindi areas, Mangochi District, Malawi [32]. Recruitment was stratified based on infants’ gestational age at birth to enroll infants born preterm (32.0–36.9 gestational weeks), early term (37.0–38.9 gestational weeks), and full term (39.0–41.9 gestational weeks). Infants took part in eye tracking tasks at the chronological age of 9 months (±14 days) and development assessments at the age of 18 months (±1 month). Anthropometrics and background data were collected between the enrollment and 18 months of age.

We conducted the study in accordance with the ethical standards of the Helsinki declaration. The study protocol was approved by the College of Medicine Research and Ethics Committee, Malawi; the Ethics Committee of Pirkanmaa Hospital District, Finland; and the Ethics Committee of the Tampere Region, Finland. A written informed consent was obtained from a parent or legal guardian on behalf of the participants at the enrollment and before the 18-month-visit.

### Eye-tracking-based assessment of attention at 9 months

**Fig 1:**
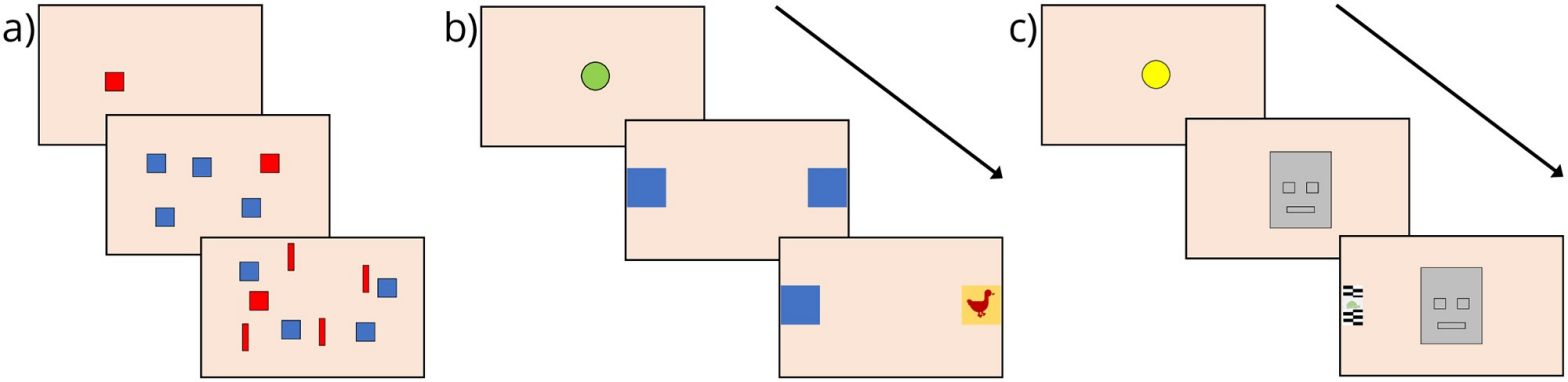
Illustrative images of the eye tracking tasks. a) Three conditions of the visual search task. b) Sequence of the anticipatory attention shifts task. c) Sequence of the attention to faces task.

Infant’s cognitive development at 9 months of age was measured with three eye-tracking-based tasks (Fig 1) generating measures in four domains of early attentional capacities: visual search latency, visual search in the context of interfering stimuli, anticipatory attention shifts, and attention to faces. Details of these assessments are provided in Pyykkö *et al*. [32].

Infants were seated in front of a 22-inch monitor which displayed the tasks and their gaze was tracked with a remote Tobii X2-60 eye tracker (Tobii Technology, Stockholm, Sweden). Gaze data were recorded on 60 Hz and consisted of the onset times of images, *xy*-boundaries of active areas of interest on the screen, and *xy*-coordinates with validity estimates of the participants’ point of gaze.

After calibration, the three tasks were performed twice with a break between two sessions.

#### Calibration

Calibration had five points (cartoon images in four corners and the center of the screen) which appeared one at a time, after the participant moved their gaze into one. Calibration was done a maximum of three times to achieve a satisfactory calibration. Assessor rated the calibration as “good”, “OK”, “poor”, or “invalid” by comparing the visualization of the calibration outcome to predefined criteria.

#### Visual search

In *the visual search task* (based on [26]), we measured infants’ reaction time to move their gaze to a salient visual target (a red apple). As described in Pyykkö *et al*. [32], the task started with an “oh” sound and the presentation of an image of a red apple (5 visual angle) on the center of the screen. After the infant looked at the apple and 2,000 ms elapsed (or a maximum wait period of 4,000 ms elapsed), the apple was removed for 500 ms and subsequently reappeared in a randomly chosen location on the screen. Depending on an experimental condition, the apple reappeared either alone (*one-object condition*), together with four or eight distractors of one kind (e.g., four blue apples or four rectangle-shaped sliced apples, *multiple-objects condition*), or together with four or eight distractors of two kinds (e.g., two/four blue apples and two/four red sliced apples, *conjunction condition*). When the infant’s point of gaze hit the target or 4,000 ms elapsed from the start of the trial, the target made a spinning movement on the screen and a reward sound was played. There were four trials per condition in one session, i.e., 24 trials in total. A blank screen was presented for 500 ms between trials.

#### Anticipatory attention shifts

In *the anticipatory attention shifts task* (adapted from [14]), we examined infant’s ability to anticipate the appearance of a visual stimulus in a predictable location [32]. The infants were presented first with an attention-getting stimulus (a pink pig face, 5 visual angle) in the center of the screen. When the infant’s gaze hit the central stimulus, the stimulus was removed and an auditory cue was presented together with two empty rectangles on both sides of the screen. A reward image (an animated duck) was subsequently shown in one of the two rectangles. The reward was presented on the same side (counterbalanced left or right) during the first eight trials (*pre-switch*). The side was then switched for the last eight trials (*post-switch*). There were a total of 16 pre-switch and 16 post-switch trials in two sessions. The time interval from the presentation of the two empty rectangles to the presentation of the rewards was contingent on the participant’s behavior. If the infant made a “correct” anticipatory saccade to the placeholder where the reward stimulus was about to appear, the reward was presented without a delay. If there was no correct anticipatory saccade, the reward was presented after a 1,000-ms delay.

#### Attention to faces

*The attention to faces task* was an overlap paradigm in which infant’s dwell time on a central stimulus (a non-face pattern or a face) was measured before its shift to a lateral distractor (following [29, 30, 46–48]). Each trial started with an attention-grabbing stimulus in the center of the screen. After a fixation at this stimulus, the attention-grabber was removed and two new stimuli were presented with a 1,000-ms onset asynchrony. First, a non-face pattern or a face on the center, then on the left or right side of the screen a black and white geometric shape superimposed by a cartoon. When the infant’s gaze moved to the lateral image or 1,000 ms elapsed, the cartoon picture turned into a video animation. The faces were pictures of two Black females with happy and fearful expressions. The non-face patterns were rectangular and phase-scrambled from the faces. Trials were presented in a random order and consisted of eight non-face trials and eight face trials (four happy and four fearful) per session (16 non-face trials and 16 face trial in total).

#### Extraction of key variables

Raw eye tracking data were preprocessed and analyzed offline by using a library of automated MATLAB (The MathWorks Inc.) functions [31]. The analyses followed the approach described in Pyykkö *et al*. [32] and no changes to the analyses of the eye tracking data were made for the current association analyses. The *xy*-coordinates corresponding to the two eyes were combined by taking a mean of the coordinates (or by using the eye with valid *xy*-coordinates if one of the coordinates for one of the eyes was invalid), extrapolated to fill missing data points (maximum of 200 ms), and median filtered with a moving window of nine samples to remove abrupt technical spike artefacts from the data. In each task, trials that failed to meet predetermined data quality criteria (i.e., violated upper limit of extrapolation) were excluded. Additional task-specific exclusion criteria were applied for the assessment of attentional dwell times in the face task so that trials with < 70% fixation on the central stimulus prior to attention shift and trials on which the shift occurred during a period of extrapolated data were excluded.

From the visual search task, we extracted the visual search latency by calculating the mean latency of gaze shifts that entered the target area within a time period that started 150 ms after the onset of the target and ended 850 ms later. These limits are based on the convention that orienting responses shorter than 150 ms are considered anticipatory and orienting responses longer than 1,000 ms as delayed or missing responses. The search latencies were calculated by using data from the one-object condition because this condition was the only one that had a high rate of successful responses in all participants (mean 92%, range 20–100%) and because previous studies assessing infant’s processing speed as a predictor of cognitive development have used comparable measures [6, 7]. The latencies were calculated from a maximum of 8 trials per participant. Given variable number of successful search responses in the other (multi-object) conditions of the visual search task, the proportion of successful search responses (instead of latency) was calculated to obtain performance indicators for the multiple-objects and conjunction conditions. These indicators were calculated by counting the number of trials on which the point of gaze entered the target area within 2,000 ms and dividing the count by the total number of valid trials.

From the anticipatory attention shifts task, we extracted the proportion of anticipatory gaze shifts to the correct side of the reward stimulus. As described in Pyykkö *et al*. [32], anticipatory responses were defined as entries of the point of gaze in the correct area of interest (i.e., side of the reward stimulus) within a 1,150-ms time window that started at the onset of the two empty rectangles and ended 150 ms after the onset of the reward stimulus. Again, the time window was extended to 150 ms after the onset of the reward stimulus on the basis of the commonly held assumption that gaze shifts that are shorter than 150 ms are typically considered anticipatory and could not, therefore, be reflecting a reactive saccade to the reward. Anticipatory gaze shifts were analyzed separately for the pre-switch and post-switch conditions. Trial numbers 1, 9, 17, and 25 were excluded from pre- and post-switch success rates as they were not predictable. Thus, for each condition, there was a maximum of 14 trials per participant.

The data from the tasks assessing attention to faces was analyzed by computing the duration the infant gaze dwelled in the center area of interest (AOI) before an attention shift to the peripheral AOI occurred [32]. The analyses were censored at 3,500 ms meaning that if no attention shift occurred before this time-out value, the dwell time was 3500 ms. The dwell times < 150 ms were excluded. Dwell times were calculated separately for non-face patterns and faces. Both conditions had maximum of 16 trials by participant.

For a participant to be included in the analyses of four visual attention measures, the participant needed to provide at least three valid trials for each condition of the task for a particular measure. For sensitivity analysis, as an overall visual attention score, each variable (excluding dwell time on faces, as its direction cannot necessary be defined as positive or negative) was ranked by quickness or success. Participant’s percentile on the seven conditions were summed.

### Development assessment at 18 months

We assessed four domains of child development at 18 months of age. First, at the home visit child’s mother or caregiver was asked about the child’s word usage and socioemotional behavior. Later, at the clinic visit, the child was assessed on gross and fine motor development and executive functioning. Each assessment was performed once. Children were not asked for a re-visit if the assessment was unsuccessful. The development assessment tasks were previously used in the same location [49].

#### Language

Child’s language development was assessed using a 100-word local language vocabulary checklist [50], which was translated into Chichewa and Chiyao and adapted for use in Malawi [49]. The mother or caregiver was presented with words from an existing list of words and asked to indicate whether the child has said the word. The child’s score was the cumulative total of positive responses (*language score*, maximum of 100).

#### Socioemotional development and maladaptive behavior

Child’s socioemotional development was assessed by using the Profile of Social and Emotional Development. The questionnaire was based on the Child Behaviour Questionnaire for Parents [51]. The mother or caregiver was asked 19 open-ended questions regarding problems in different domains of behavior (e.g., self-help, independence, attention, aggression, following rules, emotion control, playing with others, trouble in adjusting to changes), and the assessor rated answers as 0, 1 or 2 based on seriousness and frequency of the problem. Additionally, child’s maladaptive behavior was assessed by asking 11 open-ended questions regarding possible unusual or rare behaviors (e.g., showing no affection, unresponsiveness). The answers were scored as a binary variable where 0 indicate the absence of the behavior and 2 regular behavior. The sum score of the questionnaire across all 30 questions was reversed for higher score to indicate fewer socioemotional problems (*socioemotional score*, maximum of 60).

#### Motor development

Child’s motor development was assessed with the Kilifi Developmental Inventory tool developed in Kenya [52]. The assessment consisted of 36 gross motor (e.g., jumps with two feet, climbs onto platform; and an extra item of running) and 59 fine motor (e.g., kicks a ball, can do up button, puts coins in a box) items scored as binary variables (1 for successfully completed items). Some items were progressive, i.e., successful completion of the previous items was a requirement for the administration of the item (e.g., building tower of blocks was scored on a two-block interval, maximum of twelve blocks). A composite *motor score* was created by summing across all 95 items (max. score 95).

Child’s mood, activity level, and interaction with the assessor were also rated. Mood was scaled from crying to laughing, activity level from unrousable to active, and interaction from avoidant to friendly.

#### Executive functioning

After the assessment of motor skills, the child’s executive function and working memory was assessed using a version of the A-not-B task [16]. Two cups were placed on a board in front of the child and the child saw a treat (a piece of corn puff) being put under either of the cups. The board with the two cups was subsequently hidden for 5 seconds and the child was distracted. When the board was returned, the child was asked to select the cup hiding the treat. The place of the treat was switched after two consecutive successful selections. The task was repeated ten times. Successful selections after a switch of a location were scored as 1 and summed (*A-not-B score*, maximum of 4). Only participants with the full ten attempts were included in analyses.

### Growth

Data on the child’s length, weight, head circumference, and mid-upper arm circumference were collected at enrollment, 9, and 18 months. Age- and sex-standardized anthropometric indices (length-for-age, LAZ; weight-for-age, WAZ; weight-for-length, WLZ; head circumference-for-age, HCAZ; and mid-upper arm circumference-for-age *z*-scores, MUACZ) were calculated using World Health Organization Child Growth Standards [53]. MUACZ was not available for enrollment measurements and thus, MUAC adjusted to age and sex was used instead when enrollment measurements were used. The change in anthropometric indices between 9 and 18 months of age (Δ9–18) was calculated as a measure of relative growth velocity adjusting for genetic potential which describes environmental factors affecting growth [54].

### Maternal and family data

Mothers and family were assessed between the enrollment and 9 months on constructs of *maternal cognition*, *maternal psychosocial well-being*, *socioeconomic status*, and *care practices*. Details of these assessments are provided in Pyykkö *et al*. [32].

In the tests of maternal cognition, mothers were asked to complete tests assessing spatial cognition (mental rotation), working memory (digit span forward and backward test), and verbal fluency (listing foods and girls’ names). The tests of mental rotation consisted of five rows, one kind a figure in each row. Following an example figure, the mother was asked to point out rotated, not flipped figures. In the digit span tests, mothers were asked to repeat sequences of digits, first in the same order as presented and then in a reversed order. In both sets, the length of the sequence increased on every trial. In the listing tests, mothers were given 60 seconds to name as many foods as possible, then another 60 seconds to name as many girls’ names as possible.

Maternal psychosocial well-being construct was assessed by questionnaires covering depression symptoms [55, 56], perceived stress [57], life events, and social support. Answer options were either yes/no or on different scales (numerical input from 0 to 2–4). Scores were summed within each subscale.

Socioeconomic status was assessed on the basis of responses to questions concerning food insecurity in family (access to food, hunger) [58], living conditions (house material and equipment, water source), and the satisfaction of everyday needs (questions about money, food, laundry).

Care practices consisted of an observation at home [59] and questions related to mother-infant bonding [60]. Observations and questions targeted mothers’ interaction styles and activities promoting cognitive, motor, and socioemotional development of the child. Mother-infant bonding consisted of questions about mother’s feelings toward the child, as described with different adjectives (a 4-point scale).

Each variable was standardized and then summed within a domain to construct these four domains. Higher score indicates positive or better responses.

### Data analysis

We carried out the statistical analyses with Stata 15.1 (StataCorp) and R 4.0.0 (R Core Team). Graphics were produced with Microsoft PowerPoint (Microsoft Corporation) and R’s ggplot2 library [61].

We used correlation coefficients to examine associations between variables, and to allow for a clear, unified interpretation of the strengths of the associations between all comparisons. As some of the variables deviated from normal distribution, we used nonparametric methods: Spearman’s rank correlation coefficients for un-adjusted tests and Spearman’s partial rank correlation coefficients for adjusted tests (adjustments are listed inside parenthesis in the following chapters). We considered correlation coefficients > ∣0.20∣ significant following previous association analyses in infants.

The sample size varied between analyses (*n* = 198–377) depending on the availability of valid data. For the constructs of the 9-month developmental scores, growth, and family characteristics, with the smallest sample size of a construct (*n* = 198, 254, and 262, respectively) and using a Bonferroni adjustment for the number of tests in each family of hypothesis tests (*n* = 16, 44, and 16, respectively), the two-sided *p*-values for the correlation coefficient ∣0.20∣ are < 0.08 (*p* = 0.076, 0.060, and 0.018, respectively).

In the main analyses, we calculated correlation coefficients between measures of infant attention at 9 months and the developmental outcomes at 18 months. When necessary, the measure of interest was adjusted for related, but non-critical variability in task performance by using partial correlation tests (e.g., the proportion of successful visual searches in the context of interfering stimuli was adjusted for general proportion of successful visual searches in conditions that did not have the interfering elements). We focused on the four constructs that the eye tracking tasks were designed to measure at 9 months of age: (a) visual search latency using data from the one-object condition, (b) visual search interference (i.e., the proportion of successful visual searches using data from the conjunction condition; adjusted to the proportion of successful searches in one-object and multiple-objects conditions), (c) the ability to update anticipatory attention shifts after a change in stimulus contingency (i.e., proportion of correct anticipatory responses on post-switch trials; adjusted to proportion of correct anticipatory responses on pre-switch trials), (d) average dwell time for faces (adjusted to average dwell time for non-face patterns). The development outcomes at 18 months of age consisted of the four constructs measuring (e) language, (f) socioemotional behavior, (g) motor development (adjusted to child’s behavior during the test), and (h) A-not-B score. To obtain a variable reflecting the child’s behavior during the test, the mood, activity level and interaction with the assessor were ranked and combined to one variable, extracted from the first component of a principal component analysis.

In secondary analyses, associations between growth and developmental outcomes were examined by linking (i) gestational age at birth, (j) anthropometric measurements (*z*-scores for length, weight, head circumference, mid-upper arm circumference) at 9 months of age (adjusted to the measurement at enrollment), and (k) change in anthropometric measurements between 9 and 18 months of age (adjusted to the measurement at 9 months of age) to the developmental outcomes at 18 months. In addition, we examined associations between family characteristics using (l) maternal cognition, (m) maternal psychosocial well-being, (n) socioeconomic status, and (o) care practices and the developmental outcomes at 18 months.

## Results

### Sample

**Fig 2:**
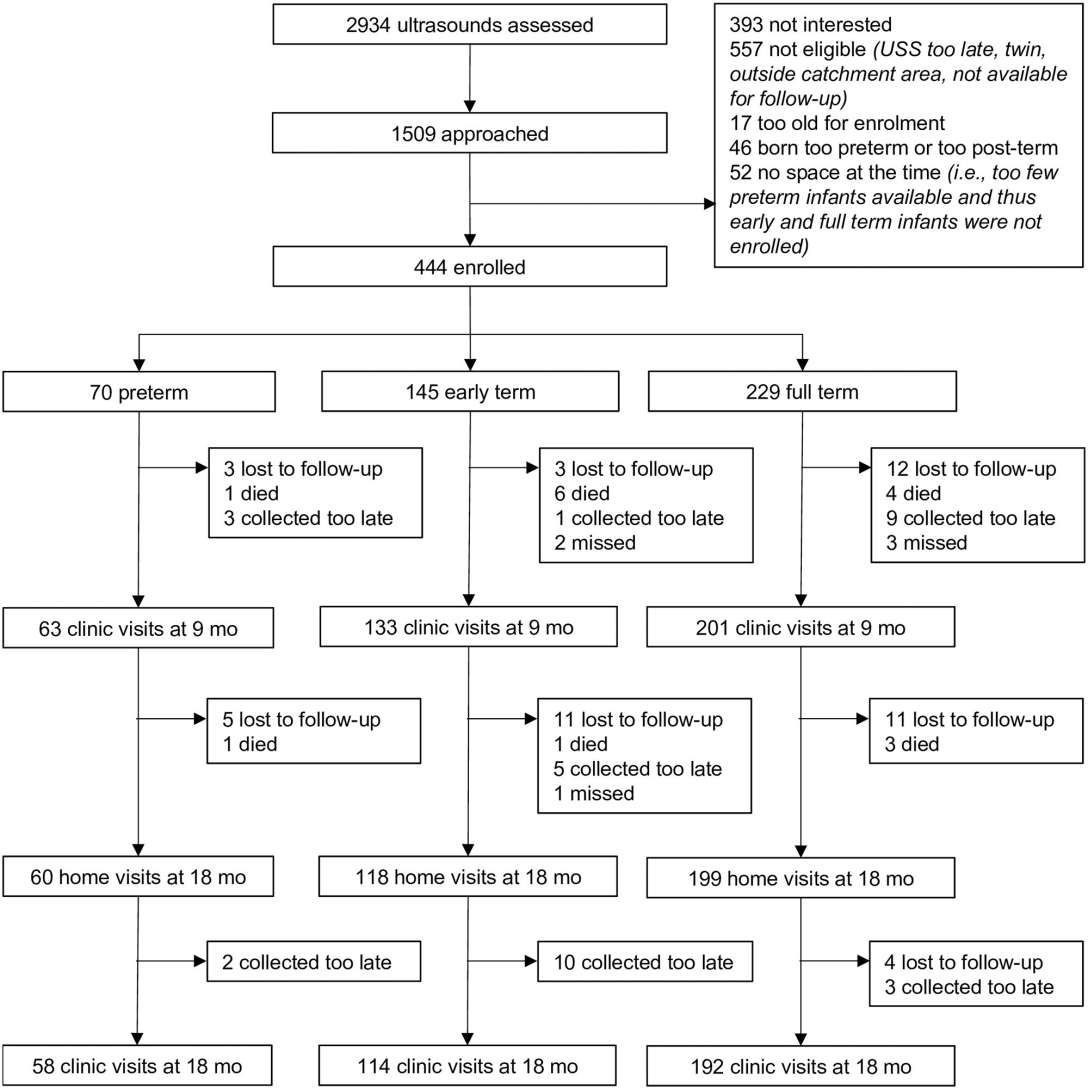
Flow chart of the study.

Between May 2016 and April 2017, a total of 377 infants were seen at the home visit, and a total of 364 infants (82.0% of enrolled) at the clinic visit at the age of 18 months (±1 month) (Fig 2). Compared to those who were enrolled but not seen at the clinic at 18 months of age, there was no significant difference in child’s anthropometrics, maternal age, maternal literacy at enrollment, or visual attention scores at 9 months of age (Table A in S1 File).

In the sample seen at 18 months, mothers had mean (*SD*) 3 (2) children, ranging from 1 to 11 children. Maternal and paternal literacy rates at enrollment were 34.7% and 61.3%, respectively (cf., census data for rural Malawians: 59.8% and 77.7%, respectively [62]). In total, 2.2% of households were in an electric grid. Scores of maternal and family data on maternal cognition, maternal psychosocial well-being, socioeconomic status, and care practices are shown in Table B in S1 File.

A total of 343 participants who were seen at 18 months were tested with eye tracking at the 9 months of age, and 318 (92.7%) of these completed all three tests. The participants had 78.4%, 72.5%, and 63.1% valid trials in total, for the visual search, the anticipatory attention shifts and the attention to faces, respectively. Validity and success rates over trial sequence are shown in Fig A in S1 File. A total of 325 participants had enough valid trials in at least one of the tasks and were included in one or more of the association analyses (Table 1).

**Table 1:** Visual attention scores at 9 months of age.

		Mean (*SD*)	
Variable	*n*	Score	Valid trials
Visual search latency, ms	282	436 (63)	6.3 (1.6)
Visual search task, one-object condition,% of successful search	295	91.9 (15.3)	6.9 (1.3)
Visual search task, multiple-objects condition,% of successful search	295	61.9 (21.8)	6.7 (1.3)
Visual search task, conjunction condition% of successful search	295	45.2 (20.3)	6.3 (1.5)
Anticipatory attention shifts task, pre-switch,% of correct anticipation	313	72.0 (26.1)	11.1 (2.7)
Anticipatory attention shifts task, post-switch,% of correct anticipation	313	54.1 (27.7)	10.1 (3.4)
Attention to faces task, dwell time on non-face patterns, ms	275	410 (145)	10.2 (3.4)
Attention to faces task, dwell time on faces, ms	275	1,922 (695)	12.5 (2.8)

Mean times to complete the tasks were 2:19 min, 3:00 min, and, 3:30 min, for the visual search, the anticipatory attention shifts, and the attention to faces, respectively (Fig B in S1 File).

**Fig 3:**
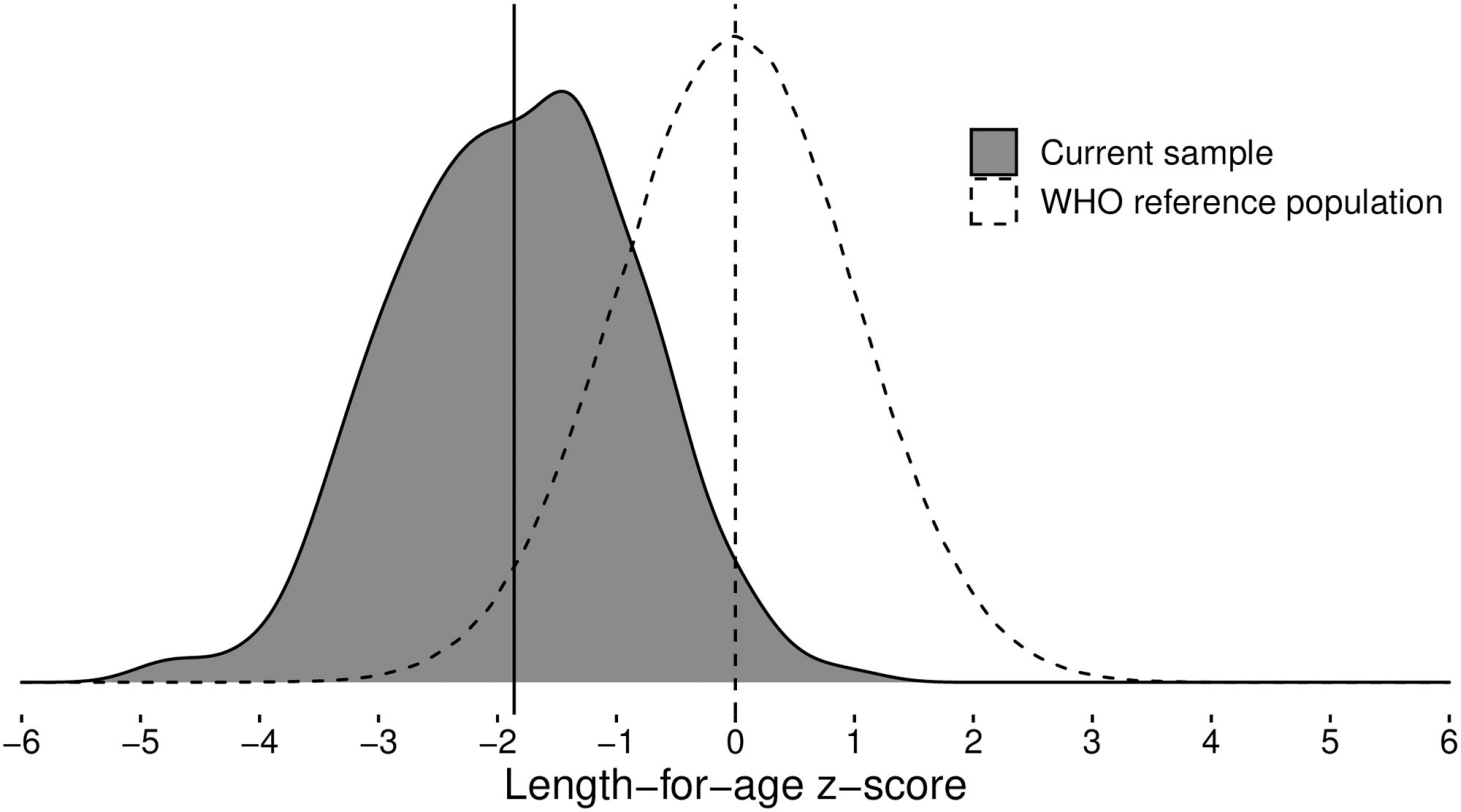
Participants’ length-for-age *z*-score distribution at the age of 18 months. Vertical lines indicate respective means.

At 18 months of age, the mean (*SD*) length and weight were 76.4 (3.0) cm and 9.5 (1.1) kg, respectively, corresponding to a mean (*SD*) LAZ of -1.86 (1.03) and a mean (*SD*) WAZ of -0.99 (0.97) (Table 2, Fig 3). Forty-four percent of children were stunted (LAZ < -2) and 15% underweight (WAZ < -2). Of the underweight children, 96% (54/56) were also stunted. The mean (*SD*) LAZ and WAZ change from 9 to 18 months of age was -0.41 (0.49) and -0.25 (0.56), respectively.

**Table 2:** Anthropometric measurements at 18 months of age.

	Mean (*SD*) or % (*n*)
Age at measurement, d	552 (9)
Length, cm	76.4 (3.0)
Weight, kg	9.5 (1.1)
Head circumference, cm	45.9 (1.4)
Mid-upper arm circumference, cm	14.7 (1.0)
LAZ	-1.86 (1.03)
WAZ	-0.99 (0.97)
WLZ	-0.13 (0.87)
HCAZ	-0.68 (0.95)
MUACZ	0.03 (0.86)
LAZ Δ9–18 months	-0.41 (0.49)
WAZ Δ9–18 months	-0.25 (0.56)
WLZ Δ9–18 months	-0.29 (0.66)
HCAZ Δ9–18 months	-0.18 (0.48)
MUAZ Δ9–18 months	0.09 (0.68)
LAZ < -2 (stunted)	44.0% (160)
WAZ < -2 (underweight)	15.4% (56)
WLZ < -2 (wasted)	1.9% (7)

*n* = 364, except for change measures *n* = 347, LAZ = length-for-age *z*-score, WAZ = weight-for-age *z*-score, WLZ = weight-for-length *z*-score, HCAZ = head circumference-for-age *z*-score, MUACZ = mid-upper arm circumference-for-age *z*-score, Δ = change. Census data for under 5-year-old: stunted 37%, underweight 12%, wasted 3% [63].

Developmental scores collected when infants were 18 months old (language, socioemotional, motor, A-not-B) were not significantly correlated with each other. The correlation coefficients varied between -0.01 and 0.09 (Table 3).

**Table 3:** Distribution and correlations between developmental scores at 18 months of age.

	Variable characteristics		Spearman’s partial rank correlation (*n*)		
	n	Mean (*SD*)	Language	Socioemotional	Motor
Language	377	32.8 (22.3)	–		
Socioemotional	377	40.8 (4.4)	0.07 (377)	–	
Motor	363	53.0 (10.6)	0.02 (362)	0.03 (362)	–
A-not-B	266	1.06 (1.10)	0.05 (266)	-0.01 (266)	0.09 (266)

Higher score indicates positive outcome.

### Associations between developmental scores and risk factors

To measure predictive associations, we calculated adjusted correlation coefficients between eye tracking measures at 9 months of age and developmental scores at 18 months of age. Correlation between measures of infant visual attention at 9 months of age and language, socioemotional, motor, and A-not-B scores at 18 months of age varied between -0.08 and 0.14 and none of them were significant or above the hypothesized level (∣0.20∣, Table 4). Relationships did not show non-linear associations or heteroskedasticity (Fig C in S1 File).

**Table 4:** Associations between developmental scores at 9 and 18 months of age.

	Spearman’s partial rank correlation (*n*)			
	Language	Socioemotional	Motor	A-not-B
Visual search latency	-0.02 (291)	0.04 (291)	0.07 (281)	-0.02 (198)
Visual search task, conjunction condition	0.01 (306)	0.03 (306)	0.14 (294)	-0.06 (210)
Anticipatory attention shifts task, post-switch	0.00 (325)	-0.08 (325)	-0.07 (312)	0.07 (226)
Attention to faces task, dwell time on faces	0.00 (283)	-0.05 (283)	0.06 (274)	-0.06 (200)

The correlations may be attenuated by low reliability of the infant measures, i.e., number of valid trials, calibration quality, and time spent on tasks. As the duration of individual test trial in most of the eye tracking was contingent on the performance (e.g., gaze entering the target area), the time spent on tasks had a negative correlation with performance indicators in many of the tasks, whereas the number of valid trials and calibration quality had no association with task performance in most of the tasks except a weak correlation with performance in some of the visual search tasks (Fig D in S1 File). To examine whether the association analyses were affected by the quality of infant measures, we adjusted the main analyses for time spent on task, number of trials, and calibration quality. The results were unchanged (Table C in S1 File). We also conducted a sensitivity analysis in which we recalculated the correlations for a subsample of infants who had the highest level of data quality based on high number of valid test trials across conditions (i.e., 8 trials on the single object condition for visual latency, 8 trials on the conjunction condition for the visual search, 13–14 trials on anticipatory attention shifts, or 15–16 trials on the attention to faces task; *n* = 45–78 depending on the test), had a good or OK calibration result, and completed all the 88 trials. In this subsample, the correlations varied between -0.22 and 0.19, but only the correlation between anticipatory attention shifts and motor score had a correlation above ∣0.20∣ (-0.22) (Table D in S1 File).

To further investigate the associations between infant attention measures and later outcomes, we examined whether a composite measure of overall visual attention performance at 9 months (created on the basis of rank order of infant performance in each of the tasks) predicted 18-month outcomes. These analyses showed that the top and bottom visual attention performers at 9 months did not have statistically significant differences in the 18-month development scores (Table E and Fig E in S1 File).

The gestational age at birth, child’s anthropometrics at 9 months of age, and anthropometric measurement changes between 9 and 18 months of age were not associated with developmental scores at 18 months of age (correlations between -0.10 and 0.19, Table 5). Neither were measures of maternal cognition, maternal psychosocial well-being, socioeconomic status, and care practices associated with the 18-month development scores (correlations between -0.04 and 0.12, Table 6).

**Table 5:** Associations between growth and developmental scores at 18 months of age.

	Spearman’s partial rank correlation (*n*)			
	Language	Socioemotional	Motor	A-not-B
Gestational age at birth	-0.04 (377)	0.00 (377)	-0.04 (363)	0.06 (266)
LAZ at 9 months	0.12 (359)	0.00 (359)	0.09 (346)	-0.08 (254)
LAZ Δ9–18 months	0.06 (346)	-0.10 (346)	0.16 (346)	-0.03 (254)
WAZ at 9 months	0.12 (359)	0.04 (359)	0.10 (346)	-0.04 (254)
WAZ Δ9–18 months	0.03 (346)	-0.07 (346)	0.19 (346)	-0.01 (254)
WLZ at 9 months	0.09 (359)	0.04 (359)	0.05 (346)	-0.03 (254)
WLZ Δ9–18 months	0.06 (346)	-0.05 (346)	0.16 (346)	0.00 (254)
HCAZ at 9 months	0.11 (359)	0.06 (359)	0.08 (346)	-0.07 (254)
HCAZ Δ9–18 months	0.07 (346)	0.05 (346)	0.05 (346)	-0.03 (254)
MUACZ at 9 months	0.12 (359)	0.04 (359)	0.10 (346)	-0.02 (254)
MUACZ Δ9–18 months	0.10 (346)	-0.02 (346)	0.15 (346)	0.00 (254)

LAZ = length-for-age *z*-score, WAZ = weight-for-age *z*-score, WLZ = weight-for-length *z*-score, HCAZ = head circumference-for-age *z*-score, MUACZ = mid-upper arm circumference-for-age *z*-score, Δ = change.

**Table 6:** Associations between family characteristics and developmental scores at 18 months of age.

	Spearman’s partial rank correlation (*n*)			
	Language	Socioemotional	Motor	A-not-B
Maternal cognition	0.02 (377)	0.02 (377)	0.12 (363)	-0.01 (266)
Maternal psychosocial well-being	-0.04 (371)	0.07 (371)	0.00 (357)	0.12 (262)
Socioeconomic status	0.08 (376)	0.03 (376)	0.10 (362)	0.03 (266)
Care practices	-0.03 (375)	-0.04 (375)	0.07 (361)	-0.04 (266)

Overall, only 4% (3/76) of the calculated correlations (excluding sensitivity analyses) were above ∣0.15∣ and none was above ∣0.20∣ (all related to growth and motor scores). As an additional sensitivity analysis, we examined whether non-stunted and stunted children varied in developmental scores at 18 months. Stunted children had lower language (-5.5 words, 95% CI: -10.0, -0.9) and motor (-2.7 points, 95% CI: -4.6, -0.8) scores compared to non-stunted children, whereas there were no differences in socioemotional and A-not-B scores between the groups (Table 7, Fig F in S1 File). Pre-, early or full term children did not differ in developmental scores at 18 months (Table F in S1 File).

**Table 7:** Comparing developmental scores at 18 months of age between stunted (LAZ < -2) and non-stunted (LAZ ≥ -2) children.

	Mean (*SD*)			
	Stunted children	Non-stunted children	Difference (95% CI)	*p*
Language	29.1 (21.6)	34.6 (22.1)	-5.5 (-10.0, -0.9)	0.019
Socioemotional	41.0 (4.6)	40.7 (4.3)	0.3 (-0.7, 1.2)	0.572
Motor	51.1 (11.5)	54.5 (9.6)	-2.7 (-4.6, -0.8)	0.005
A-not-B	1.00 (1.05)	1.10 (1.12)	-0.10 (-0.36, 0.17)	0.485

Difference (95% CI) and *p* from linear regression with adjusting variables (full results in Appendix in S1 File).

## Discussion

In the current study, we examined associations between early-developing attentional abilities measured by eye tracking at 9 months of age and the scores in more conventional parent interview and observational tests of cognitive development at 18 months of age in a sample of rural Malawian infants. In contrast to the hypothesis, measures of visual attention at 9 months were not associated with measures of cognitive control, motor skills, language, or socioemotional behavior at 18 months. Additionally, gestational age, growth, and other risk factors (i.e., maternal cognition, maternal psychosocial well-being, socioeconomic status, and care practices) were not associated with developmental scores at 18 months of age. Below, we discuss factors that may have affected the sensitivity of the current analyses for finding significant associations between the measures, and the implications of our findings for research on early markers of cognitive development.

The main analyses showed no association between infant attention measures and 18-month cognitive outcomes. Some of the associations were anticipated on the basis of prior empirical findings and the hypothesis that performance in one domain that can be measured early in life (i.e., the efficiency of attentional orienting) may be an indicator that correlates with performance in other domain later in childhood [7, 8], whereas other associations were predicted on the basis of shared top and bottom visual attention performers’ neurocognitive mechanisms between the tasks used in the 9- and 18-month assessments (e.g., cognitive control, affecting attention-switching in infancy and A-not-B performance at 18 months [14]). Given that the analyses showed none of the expected associations, it is important to consider whether this result indicates a false negative, arising from methodological problems in measuring the targeted constructs, or a true lack of association.

There are several methodological factors that may have attenuated a true association between infant measures and later outcomes in the current analyses. The first concerns potential problems in implementing the tasks. The tasks used in the present study have been mainly used in high-resource settings, but less in low-income countries and rural populations. Also, infants in the study area are not used to viewing screens, monitors, or TVs. The possibility of major problems in the implementation of the tasks appears unlikely, however, as our pilot study has shown that the percentages of infants completing the eye-tracking tests were nearly as high in Malawi (90%) as in Finland (95%) as were the percentages of valid test trials (Malawi 68–73%, Finland 69–85%). There are similar data for the feasibility of the 18-month assessments in rural Malawi [49].

A second and potentially more serious problem affecting current analyses concerns measurement error. Specifically, even if the tasks were successfully implemented, the number of trials obtained to estimate performance in different domains of attention tend to be low in infant studies and by consequence, standard error in the measures relatively high. The reliability of the current infant measures was estimated in Pyykkö *et al*. [32] and the odd-even correlations were -0.02, 0.22, 0.55, and 0.62 for visual search task, visual search latency, attention to faces, and anticipatory attention shifts, respectively. Test-retest reliability scores for the measures at 18 months ranged from 0.65 to 0.82 [49]. Imprecision in measures attenuates the “true” correlations between variables [64]. When a correction for this attenuation was applied to the current results, the reported correlations were not remarkably amplified (range between -0.11 and 0.19, excluding correlations with the low reliability visual search task). Similarly, our subgroup analysis that controlled for or was based on infants with higher number of successful test trials and better calibration quality (and, by consequence, more precise estimates of performance) did not show significant associations between measures of infants’ attention at 9 months and cognitive outcomes at 18 months. Finally, overall performance in visual attention tasks did not show associations with later developmental outcomes. Thus, controlling for the impact of measurement error on correlations does not appear to result in stronger evidence for the hypothesized associations in the current data.

Aside from methodological factors, there may be other explanations for a lack of association between early visual attention and later development that arise from the potentially unique characteristics of early child development in low-resource rearing environments. One is that stable individual differences in cognitive function are not yet distinguishable at the age of 18 months, as assessed in the current study. In previous studies in Western populations, measures of infant attention have been linked with cognitive outcomes across early childhood, from 10 months to 11 years [6, 7, 9–12, 65]. It is possible, however, that the emergence of stable individual differences may take longer in more resource-constrained environments. While highly speculative at this point, it is of note that our analyses of the within-session stability of responses in the measures of infant attention in the attention to faces task tended to show lower stability of individual differences in the Malawian samples (correlations from 0.28 to 0.53) as compared to Finnish sample (correlations from 0.45 to 0.72) [66]. If individual differences were less distinguishable to begin with in the current sample at 9 months, this may attenuate the strength of association with later outcomes.

The emergence of individual differences in cognition may be sensitive not just to genetic differences, but also on variability nutrition and other aspects of the physical and social environment (e.g., amount of stimulation [67, 68]). The current sample was comparable to census data on rural Malawian population and, thereby, likely to be representative of the target population. However, when examined in a global context, the current sample reflects a lower end of the distribution in terms of the availability of nutritional and other resources. This is shown in the figure that present the distribution of length-for-age *z*-scores in the current sample in comparison to the distribution of the respective scores in the global populations as a whole. There is, hence, the possibility that genetic differences in cognitive function may not manifest early in development or may take longer to manifest in low-resource environment due to restricted range of variability in relevant environmental factors, such as nutrition.

Secondary analyses in the current study examined whether physical growth and psychosocial factors were associated with cognitive outcomes at 18 months. These associations were hypothesized on the basis of previous studies, as nutrition status (i.e., stunting) has been associated with cognitive ability and learning capacity [69, 70]. Deficiencies in nutrition may relate to development at first two years of life when brain grows rapidly [71]. In a previous study in the same geographic area, child’s length at 6 months of age was associated with language and motor scores at 18 months of age [45]. Furthermore, infant’s height and maternal nurturance have been found to have positive correlations with later development in rural India [72]. Also, socioeconomic status has been seen to affect developmental scores in children [73]. In the current study, the nutrition status was not predictive to development in a continuous scale, although stunted children had lower scores on language and motor scales, as expected [74]. The reasons for the lack of continuous association between growth and cognitive scores in the current analyses are not known, but the results are consistent with some prior findings in the same target population. The data presented in McCoy *et al*. [75] showed no difference in cognitive and socioemotional development between urban and rural and between stunted and non-stunted 3- to 4-year-old children in Malawi.

There are several ways in which the current tasks and design can be improved in future studies to further examine the association between infant measures and cognitive development. The assessment of visual attention in infants may benefit from tasks that use more dynamic, cluttered or natural scenes, and videos (e.g. [76]). Also, given that the task used in infancy targeted attention functions, it would be important to include similar measures of attention in the endpoint assessments as well. Future studies could also include a wider set of outcomes. The present analyses were restricted to a limited set of *a priori* measures, but future studies and analyses of the current data could be extended to more detailed examination of the spatial and temporal distribution of fixations and gaze paths during the tasks (e.g. [77]). More robust estimation of individual differences would also benefit from repeated estimation of the target constructs at multiple time points and from usage of multiple tasks to assess a given construct (i.e., executive function [10]). Repeated assessment of anthropometry would also help in detecting trends in growth (e.g., recovery or regression) that may modulate association with distal cognitive outcomes [78]. Finally, eye tracking combined with EEG or heart rate to control arousal has been proposed [4].

In conclusion, infants’ visual search latency and success, anticipatory attention shifts, or attention to faces did not show association to infant’s development nine months later, at the age of 18 months in low-resource settings. These results further show that in this rural Malawian sample the gestational age, growth, and rearing environment had no association with infant’s later development. These results do not support the use of the current tests of infant visual attention as predictive measures in the Malawian setting. Further research is needed, however, to rule out the impact of low precision of these measures on association analyses, and to examine whether a longer follow-up period is needed to capture variations that become visible at later ages.

## Supporting information

S1 File Tables A–F, Figs A–F, and Appendix. (PDF)

S1 Data (XLSX)
