# Correction: Associations between individual variations in visual attention at 9 months and behavioral competencies at 18 months in rural Malawi

**DOI:** 10.1371/journal.pone.0241034

**Published:** 2020-10-15

**Authors:** The PLOS ONE Staff

## Notice of republication

An incorrect version of S1 Data was published in error. The publisher apologizes for the error. This article was republished on October 8, 2020 to correct for this error. Please download this article again to view the correct version.
