# Supplementary material for: Associations between individual variations in visual attention at 9 months and behavioral competencies at 18 months in rural Malawi
Source: PLoS One. 2020 Oct 1;15(10):e0239613. doi: 10.1371/journal.pone.0239613 (PMC7529224; doi:10.1371/journal.pone.0239613)
Supplement: S1 File — (PDF) [file pone.0239613.s001.pdf]

Associations between individual variations in visual attention at 9 months and behavioral competencies at 18 months in rural Malawi

Pyykkö J, Ashorn U, Chilora E, Maleta K, Ashorn P, Leppänen JM.

SUPPORTING INFORMATION

S1 File

PLOS ONE, 2020

Table A. Comparison of children seen and not seen at the clinic at the age of 18 months............................2

Table B. Scores of maternal and family data of participants seen at the clinic at the age of 18 months.........3

Table C. Associations between eye tracking measures at 9 and developmental scores 18 months of age. Adjusted for calibration quality, time spent on task, and number of valid trials. 4

Table D. Associations between eye tracking measures at 9 and developmental scores at 18 months of age for participants with high quality data.* 5

Table E. Comparison of the 18-month development scores by the overall performance in visual attention tasks.* 6

Table F. Comparing developmental scores at 18 months of age between children born preterm and term....7

Fig A. Proportion of valid and successful trials by trial sequence...8

Fig B. Time spent on visual attention tasks, both sessions combined..9

Fig C. Scatter plots with 9-month visual attention scores (x-axis) and 18-month development scores (y-axis)..10

Fig D. Spearman correlation coefficients between eye tracking quality indicators and performance measures..11

Fig E. Density plots for 18-month development scores between top and bottom performers in the 9-month visual attention tests...12

Fig F. Density plots for 18-month development scores between stunted and non-stunted participants........13

Appendix. The full results of regression analyses from Table 7..14

Table A. Comparison of children seen and not seen at the clinic at the age of 18 months.

	**Mean (*SD*) or %**		
	**Seen at 18 mo (*n* = 275–364)**	**Not seen at 18 mo (*n* = 37–80)**	***p* for difference***
Length-for-age *z*-score at enrollment	-1.18 (0.98)	-1.20 (0.95)	0.88
Weight-for-age *z*-score at enrollment	-0.85 (0.93)	-0.93 (0.99)	0.47
Maternal age at enrollment, years	25 (7)	24 (7)	0.77
Maternal literacy, %	34.7	39.3	0.47
Visual search latency, ms	436 (63)	429 (58)	0.48
Visual search task, % of successful search, conjunction	45.2 (20.3)	46.6 (0.21)	0.68
Anticipatory attention shifts task, % of correct anticipation, post-switch	53.7 (28.0)	57.5 (30.7)	0.41
Attention to faces task, dwell time on faces	1922 (42)	1892 (127)	0.80

* P values from t-test or Fisher's exact test

Table B. Scores of maternal and family data of participants seen at the clinic at the age of 18 months.

Variable	N	Mean (SD)	Range	Max. possible
*Maternal cognition**	364	*-0.01 (3.07)*	*-7.31, 9.85*	
Mental rotation test		24.9 (4.8)	13, 40	40
Digit span test, forward		5.2 (1.4)	2, 10	18
Digit span test, backward		2.4 (1.5)	0, 8	16
Verbal fluency test, foods		15.4 (4.7)	5, 29	*NA*
Verbal fluency test, girls' names		16.4 (5.2)	3, 31	*NA*
*Maternal psychosocial well-being**	358	*-0.03 (2.67)*	*-8.05, 6.24*	
Depression symptoms		14.1 (4.2)	2, 20	20
Perceived stress		21.9 (4.2)	9, 32	40
Adverse life events		29.4 (2.8)	20, 34	34
Social support		34.0 (7.5)	12, 48	48
*Socioeconomic status**	363	*-0.05 (2.05)*	*-5.22, 6.26*	
Satisfaction of everyday needs		5.1 (1.6)	1/3, 9	9
Food insecurity		18.5 (5.3)	3, 27	27
Living conditions		13.0 (1.9)	8, 21	*NA*
*Care practices**	362	*0.04 (1.42)*	*-4.75, 3.83*	
Mother-infant bond		18.9 (2.5)	11, 24	24
HOME observation		23.8 (2.4)	13, 30	36

* Standardized composite score of variables below.

Higher score indicates positive outcome for all variables.

Table C. Associations between eye tracking measures at 9 and developmental scores 18 months of age. Adjusted for calibration quality, time spent on task, and number of valid trials.

	**Spearman's partial rank correlation (*n*)**			
	**Language**	**Socioemotional**	**Motor**	**A-not-B**
Visual search latency	-0.03 (291)	0.04 (291)	0.08 (281)	-0.04 (198)
Visual search task, conjunction condition	-0.01 (306)	0.03 (306)	0.13 (294)	-0.04 (210)
Anticipatory attention shifts task, post-switch	-0.01 (325)	-0.08 (325)	-0.07 (312)	0.08 (226)
Attention to faces task, dwell time on faces	0.00 (283)	-0.06 (283)	0.07 (274)	-0.08 (200)

Table D. Associations between eye tracking measures at 9 and developmental scores at 18 months of age for participants with high quality data.*

	Spearman's partial rank correlation (n)			
	**Language**	**Socioemotional**	**Motor**	**A-not-B**
Visual search latency	-0.17 (78)	-0.15 (78)	-0.04 (78)	-0.19 (58)
Visual search task, conjunction condition	-0.09 (70)	-0.10 (70)	0.02 (69)	0.19 (50)
Anticipatory attention shifts task, post-switch	-0.06 (62)	-0.11 (62)	-0.22 (61)	-0.13 (45)
Attention to faces task, dwell time on faces	0.12 (77)	0.07 (77)	0.17 (76)	-0.19 (58)

* Subset of participants with OK/good calibration, all 88 trials recorded, and high number of valid trials on the particular task (i.e., 8 trials for visual search latency, 8 trials on the visual search's conjunction condition, 13–14 trials on anticipatory attention shifts' post-switch, or 15–16 trials on the attention to faces task's dwell times on faces).

Table E. Comparison of the 18-month development scores by the overall performance in visual attention tasks.*

	Mean (*SD*)		Wilcoxon rank-sum test	
	**Top visual attention performers (n = 29)**	**Bottom visual attention performers (n = 28)**	z	p
Language	29.8 (19.5)	31.7 (18.1)	0.783	0.43
Socioemotional	41.0 (4.6)	40.9 (4.3)	-0.413	0.67
Motor	56.1 (7.9)	54.4 (6.3)	-1.049	0.29
A-not-B	0.96 (0.89) (n = 25)	0.54 (0.65) (n = 26)	-1.734	0.08

* Subset of participants with OK/good calibration, all 88 trials recorded, and high number of valid trials (at least half valid trials on every condition, i.e., ≥4 trials on the visual search conditions, ≥7 trials on the anticipatory attention shifts conditions, and ≥8 trials on the attention to faces conditions). Top performers were ranked over 75th percentile and bottom performers were ranked under 25th percentile on the composite visual attention score.

Table F. Comparing developmental scores at 18 months of age between children born preterm and term.

	Mean (*SD*)			Kruskal-Wallis equality-of-populations rank test	
	Preterm (n = 40–60)	Early term (n = 80–118)	Full term (n = 146–199)	χ^2 (df=2)	*p*
Language	33.5 (23.4)	34.9 (23.1)	31.4 (21.6)	1.805	0.41
Socioemotional	40.8 (3.5)	41.0 (4.0)	40.7 (4.8)	0.264	0.88
Motor	53.6 (8.6)	51.6 (13.0)	53.7 (9.6)	0.618	0.73
A-not-B	0.95 (1.01)	0.93 (0.97)	1.16 (1.18)	1.504	0.47

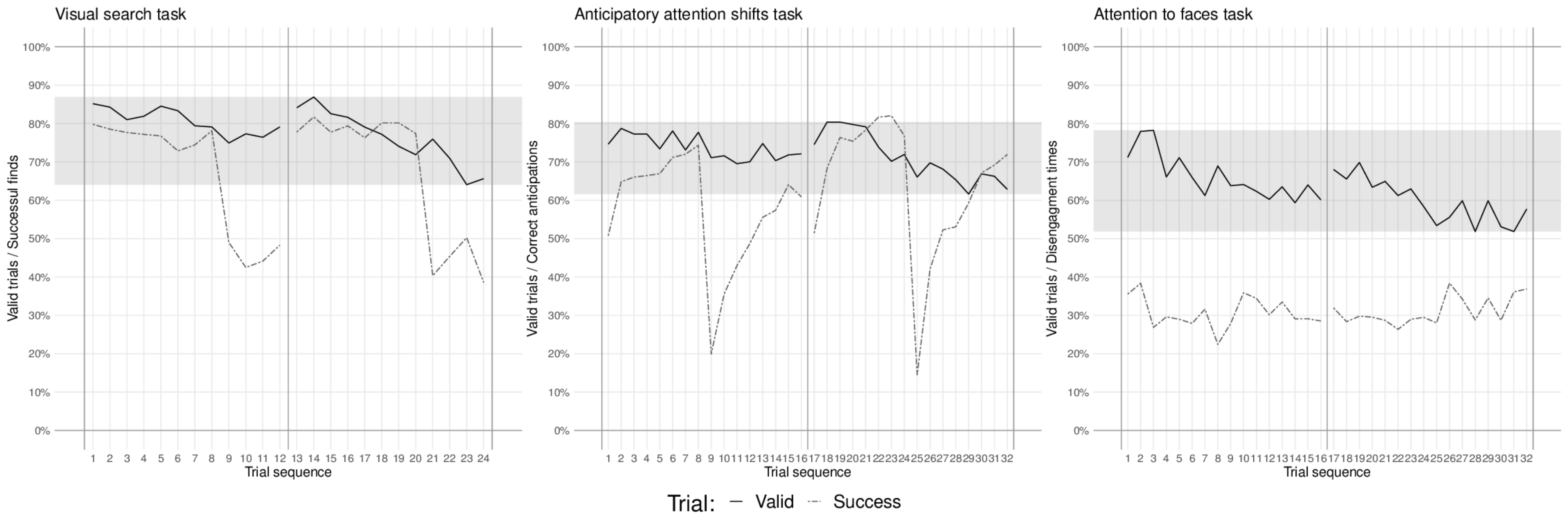

Fig A. Proportion of valid and successful trials by trial sequence.

Successful task is defined as a valid trial, in which the target was found within 2,000 ms in the visual search, as a correct anticipatory in the attention shifts task, and as a recorded or censored disengagement time (% = [mean–150] / 3,500 ms) from the central target in the attention to faces task.
In the visual search task, trials 1-8 and 13-20 were single- and multiple-object conditions and trials 9-12 and 21-24 were conjunction conditions.
In the anticipatory attention task, the target switched side at trials 9 and 25.
Gray areas define ranges, i.e., minimum and maximum, of valid trials by task within sequence. Vertical lines define session breaks.

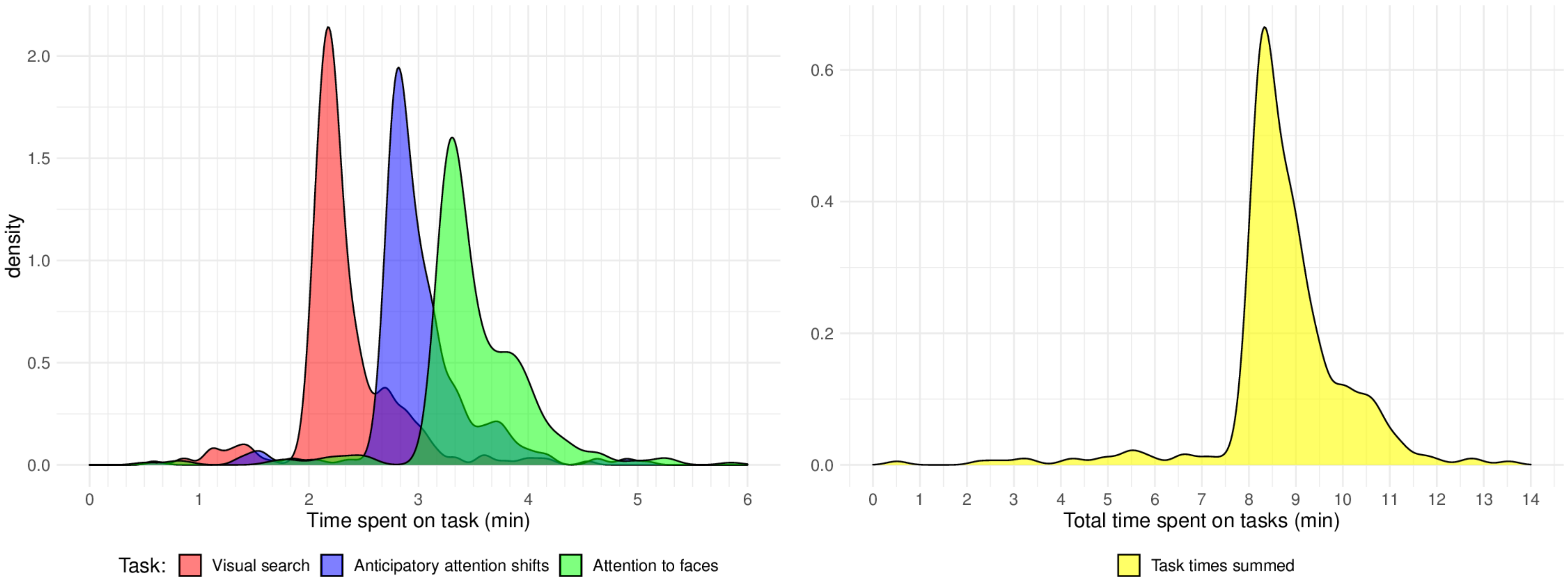

Fig B. Time spent on visual attention tasks, both sessions combined.

Task time means: 2 min 19 s, 3 min 00 s, 3 min 30 s, for Visual search, Anticipatory attention shifts, and Attention to faces, respectively, n = 340–341. Total time mean: 8 min 44 s, n = 343.

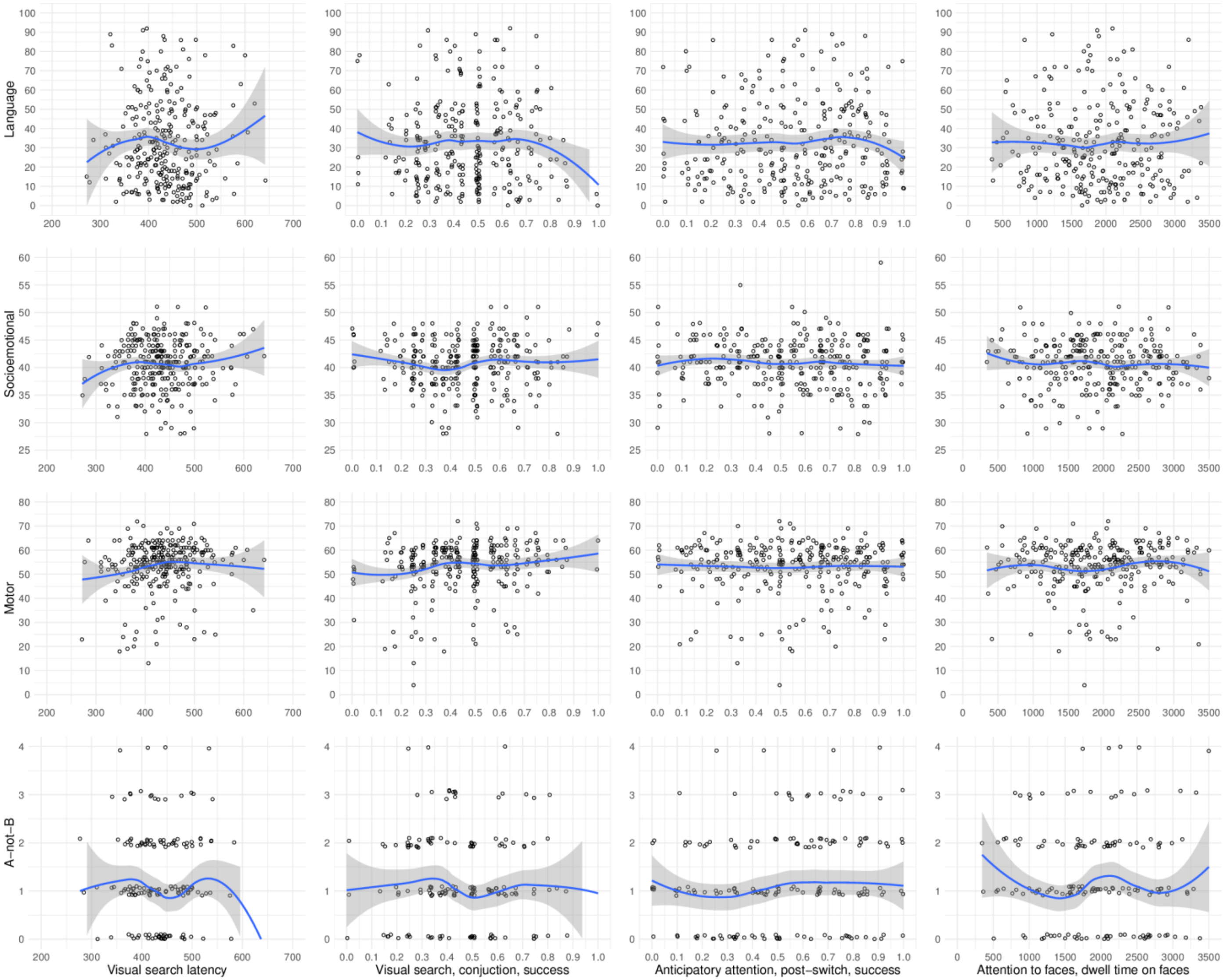

Fig C. Scatter plots with 9-month visual attention scores (x-axis) and 18-month development scores (y-axis).

Dots are jittered for visualization purposes. Blue line and gray confidence interval from Local Polynomial Regression Fitting. *Note:* Two-dimensional scatter plots ignore adjustment variables, i.e., these visuals do not relate to main comparisons in Table 4.

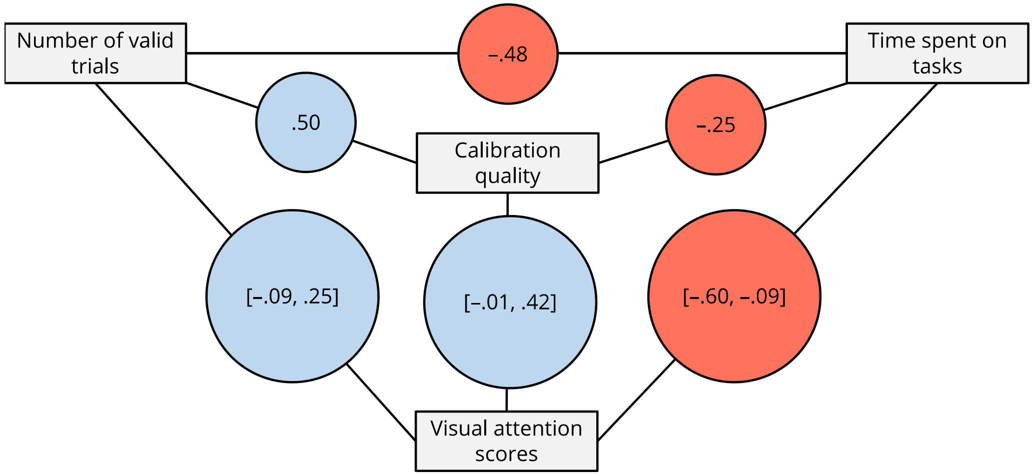

Visual attention scores	Number of valid trials	Calibration quality	Time spent on tasks
Visual search latency	0.10	0.19	-0.23
VS one-object	0.25	0.42	-0.53
VS multiple objects	0.22	0.24	-0.60
VS conjunction	0.09	0.20	-0.50
AA pre-switch	0.17	-0.01	-0.33
AA post-switch	0.13	-0.01	-0.22
AF non-face pattern	-0.09	0.09	-0.09
AF faces	-0.01	0.08	-0.10

Fig D. Spearman correlation coefficients between eye tracking quality indicators and performance measures.

The correlations are shown inside the circles between lines connecting measures (blue for positive, red for negative correlations). Correlations related to visual attention scores are expanded in the table.
Correlation coefficients with visual attention scores include eight different scores (visual search latency, three visual search conditions [VS], two anticipatory attention shifts conditions [AA], and two attention to faces conditions [AF]) and are compared against the task's number of valid trials and time spent on it.
Direction of variables: more trials, more time spent, better calibration quality, quicker responses, more successful finds, more successful anticipatory shifts, and more disengagements to lateral distractor.

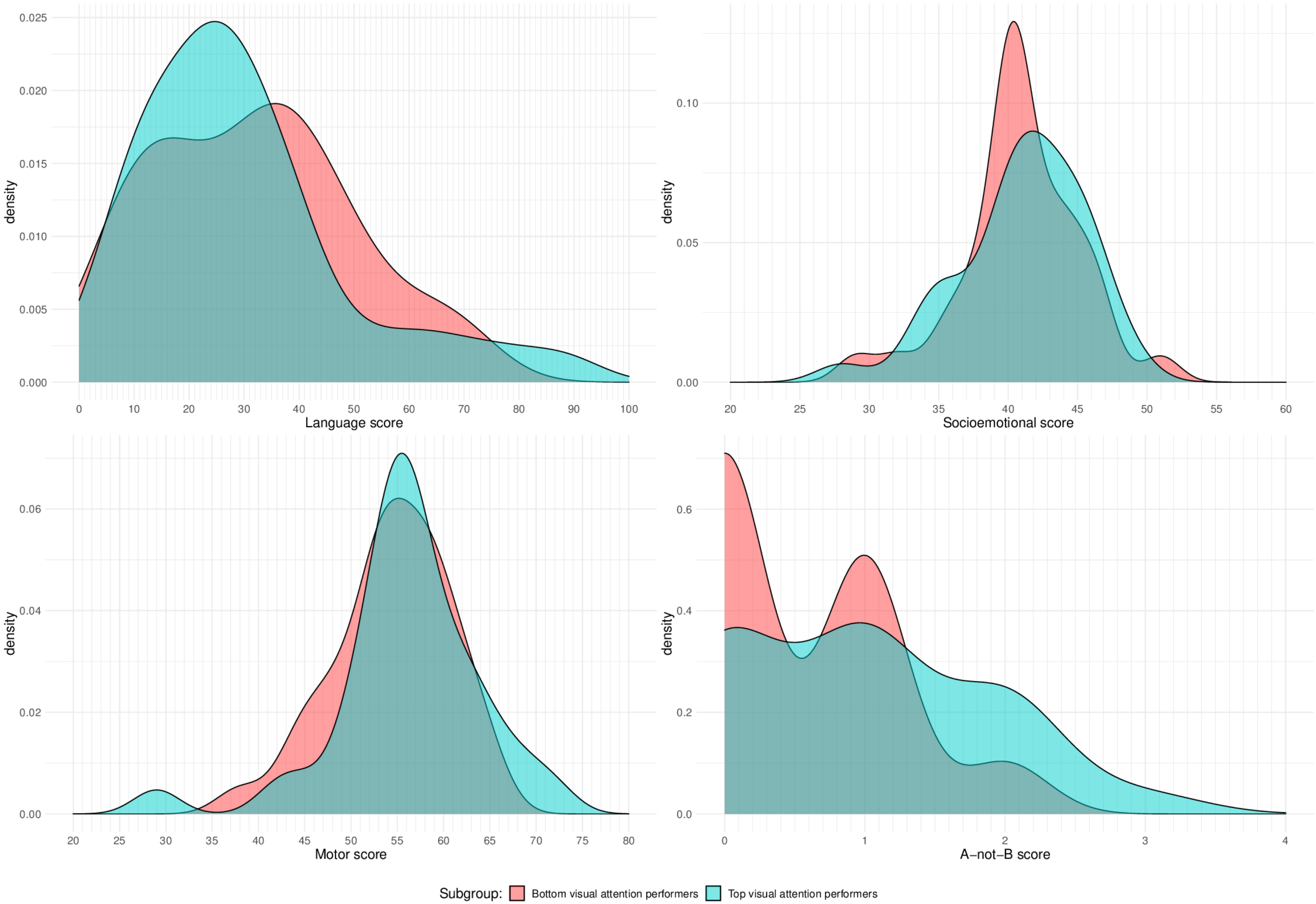

Fig E. Density plots for 18-month development scores between top and bottom performers in the 9-month visual attention tests.

Subset of participants with OK/good calibration, all 88 trials recorded, and high number of valid trials (at least half valid trials on every condition, i.e., ≥4 trials on the visual search conditions, ≥7 trials on the anticipatory attention shifts conditions, and ≥8 trials on the attention to faces conditions). Top performers were ranked over 75th percentile and bottom performers were ranked under 25th percentile on the composite visual attention score.

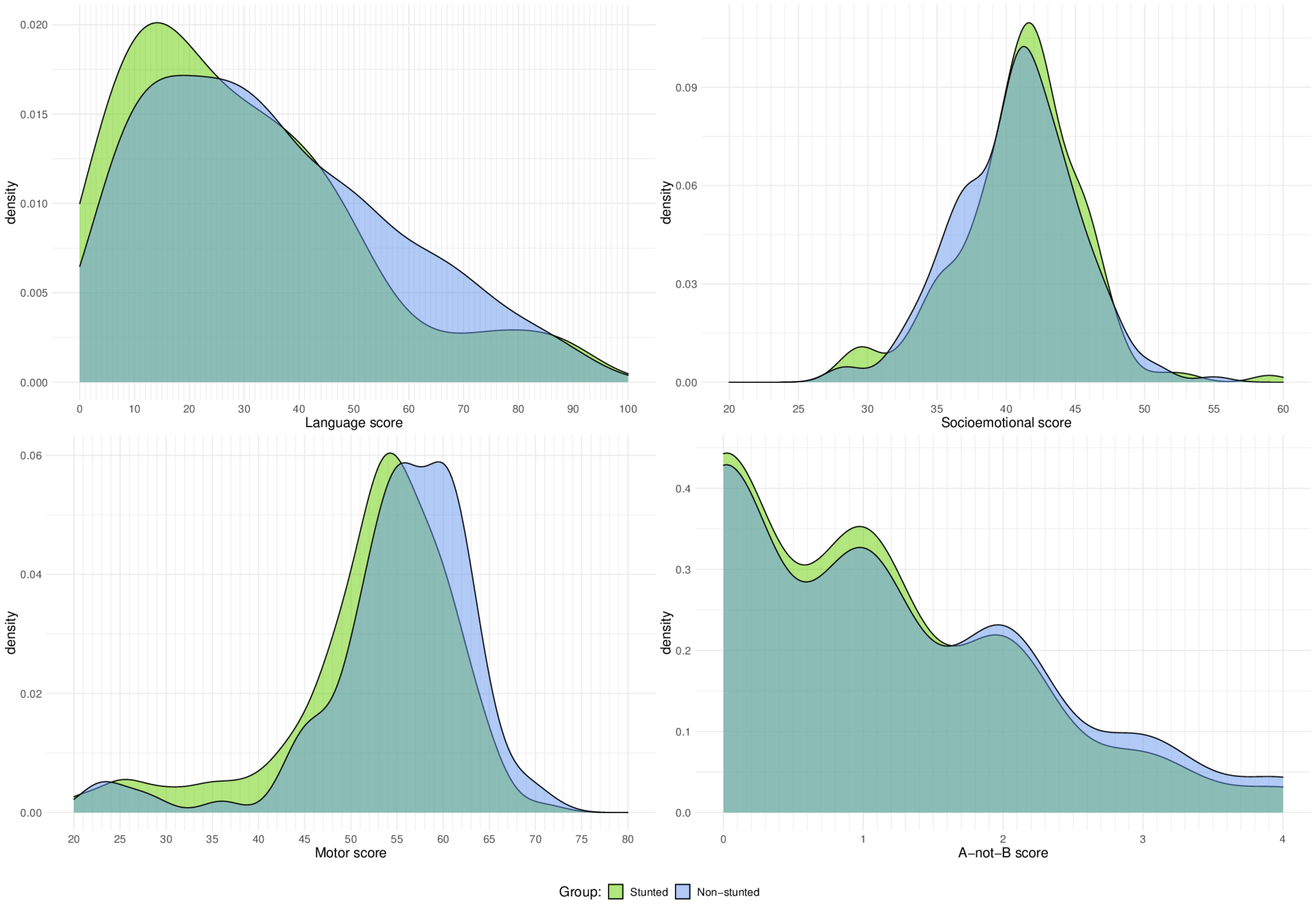

Fig F. Density plots for 18-month development scores between stunted and non-stunted participants.

Appendix. The full results of regression analyses from Table 7.

```
      Source |       SS           df       MS      Number of obs   =       363
-------------+----------------------------------   F(1, 361)       =      5.54
       Model |  2656.65492         1  2656.65492   Prob > F        =    0.0192
    Residual |  173237.229       361  479.881522   R-squared       =    0.0151
-------------+----------------------------------   Adj R-squared   =    0.0124
       Total |  175893.884       362  485.894708   Root MSE        =    21.906
------------------------------------------------------------------------------
    Language |      Coef.   Std. Err.      t    P>|t|     [95% Conf. Interval]
-------------+----------------------------------------------------------------
     Stunted |  -5.452645    2.31743    -2.35   0.019       -10.01   -.8952862
       _cons |   34.57843   1.533741    22.55   0.000     31.56224    37.59462
------------------------------------------------------------------------------


        Source |       SS           df       MS      Number of obs   =       363
-------------+----------------------------------   F(1, 361)       =      0.32
         Model |  6.33126034         1  6.33126034   Prob > F        =    0.5717
      Residual |  7132.93045       361  19.7588101   R-squared       =    0.0009
-------------+----------------------------------   Adj R-squared   =   -0.0019
         Total |  7139.26171       362  19.7217174   Root MSE        =    4.4451
------------------------------------------------------------------------------
Socioemotional |      Coef.   Std. Err.      t    P>|t|     [95% Conf. Interval]
-------------+----------------------------------------------------------------
       Stunted |  .2661857   .4702405     0.57   0.572    -.6585691    1.190941
         _cons |  40.69608   .3112184   130.76   0.000     40.08405    41.30811
------------------------------------------------------------------------------


      Source |       SS           df       MS      Number of obs   =       363
-------------+----------------------------------   F(2, 360)       =     71.58
       Model |  11663.8638         2   5831.9319   Prob > F        =    0.0000
    Residual |  29330.9131       360  81.4747585   R-squared       =    0.2845
-------------+----------------------------------   Adj R-squared   =    0.2805
       Total |  40994.7769       362   113.24524   Root MSE        =    9.0263
------------------------------------------------------------------------------
       Motor |      Coef.   Std. Err.      t    P>|t|     [95% Conf. Interval]
-------------+----------------------------------------------------------------
     Stunted |  -2.729778   .9570149    -2.85   0.005     -4.61182   -.8477356
    Assessor |   3.602628   .3159118    11.40   0.000     2.981363    4.223892
       _cons |   54.16975   .6327999    85.60   0.000      52.9253     55.4142
------------------------------------------------------------------------------


      Source |       SS           df       MS      Number of obs   =       266
-------------+----------------------------------   F(1, 264)       =      0.49
       Model |  .587256357         1  .587256357   Prob > F        =    0.4853
    Residual |  317.566879       264  1.20290484   R-squared       =    0.0018
-------------+----------------------------------   Adj R-squared   =   -0.0019
       Total |  318.154135       265  1.20058164   Root MSE        =    1.0968
------------------------------------------------------------------------------
     A-not-B |      Coef.   Std. Err.      t    P>|t|     [95% Conf. Interval]
-------------+----------------------------------------------------------------
     Stunted |  -.0955414   .1367393    -0.70   0.485    -.3647798     .173697
       _cons |   1.095541   .0875318    12.52   0.000     .9231921    1.267891
------------------------------------------------------------------------------
```
